# Combined Pituitary Hormone Deficiency in *lhx4*-Knockout Zebrafish

**DOI:** 10.3390/ijms25137332

**Published:** 2024-07-04

**Authors:** Nicole Roisman-Geller, Odelia Pisanty, Alon Weinberger, Deodatta S. Gajbhiye, Matan Golan, Yoav Gothilf

**Affiliations:** 1 School of Neurobiology, Biochemistry and Biophysics, Faculty of Life Sciences, Tel-Aviv University, Tel-Aviv 6997801, Israel; nicoler@mail.tau.ac.il (N.R.-G.); odeliap@post.tau.ac.il (O.P.); alon.weinberger@mail.huji.ac.il (A.W.); 2 Department of Poultry and Aquaculture, Institute of Animal Sciences, Agricultural Research Organization, Volcani Center, Rishon Letziyon 7505101, Israel; deodattagajbhiye@gmail.com (D.S.G.); matang@volcani.agri.gov.il (M.G.); 3 Sagol School of Neuroscience, Tel-Aviv University, Tel-Aviv 6997801, Israel

**Keywords:** *Lhx4*, zebrafish, CPHD, pituitary, development

## Abstract

LIM homeobox 4 (LHX4) is a transcription factor crucial for anterior pituitary (AP) development. Patients with *LHX4* mutation suffer from combined pituitary hormone deficiency (CPHD), short statures, reproductive and metabolic disorders and lethality in some cases. *Lhx4*-knockout (KO) mice fail to develop a normal AP and die shortly after birth. Here, we characterize a zebrafish *lhx4*-KO model to further investigate the importance of *LHX4* in pituitary gland development and regulation. At the embryonic and larval stages, these fish express lower levels of *tshb* mRNA compared with their wildtype siblings. In adult *lhx4*-KO fish, the expressions of pituitary hormone-encoding transcripts, including *growth hormone* (*gh)*, *thyroid stimulating hormone* (*tshb)*, *proopiomelanocortin* (*pomca)* and *follicle stimulating hormone* (*fshb)*, are reduced, the *pomca* promoter-driven expression in corticotrophs is dampened and *luteinizing hormone (lhb)*-producing gonadotrophs are severely depleted. In contrast to *Lhx4*-KO mice, Lhx4-deficient fish survive to adulthood, but with a reduced body size. Importantly, *lhx4*-KO males reach sexual maturity and are reproductively competent, whereas the females remain infertile with undeveloped ovaries. These phenotypes, which are reminiscent of those observed in CPHD patients, along with the advantages of the zebrafish for developmental genetics research, make this *lhx4*-KO fish an ideal vertebrate model to study the outcomes of *LHX4* mutation.

## 1. Introduction

Located underneath and regulated by the hypothalamus, the pituitary (hypophysis) functions as a master endocrine gland, relaying information from the brain to the body, thereby regulating various physiological processes, such as metabolism, growth, the stress response and reproduction. Five main types of hormone-secreting cells are present in the anterior pituitary (AP): corticotrophs that secrete the adrenocorticotrophic hormone (ACTH), which, by inducing glucocorticosteroid production in the adrenal cortex, plays a crucial role in the stress response and carbohydrate metabolism [1]; thyrotrophs that secrete the thyroid-stimulating hormone (TSH), which stimulates the synthesis and release of the thyroid hormones (THs) triiodothyronine (T3) and thyroxine (T4), thereby affecting development, growth and metabolism [2]; somatotrophs that secrete the growth hormone (GH), which stimulates growth and affects glucose metabolism [3]; lactotrophs that secrete prolactin (PRL), which stimulates milk production in mammals and controls osmoregulation in fish [4]; and gonadotrophs that secrete the gonadotropin follicle-stimulating hormone (FSH) and luteinizing hormone (LH), the key regulators of reproduction [5].

The fates of these various endocrine cell types are determined by several secreted developmental factors that create an opposing gradient along the forming AP, leading to the particular spatial expressions of a series of transcription factors, which, in turn, control the cells’ identities [6]. Mutations in such transcription factors affect the AP development, pituitary cell differentiation and pituitary hormone levels and consequently lead to broad systemic effects [7,8].

LIM homeobox 4 (LHX4) is a member of the LIM-homeodomain protein family, which bind to DNA through their characteristic helix–turn–helix motive and act as master transcription factors that regulate the gene expression involved in cell differentiation, affecting the body pattern formation during embryonic development, including the development of the endocrine and nervous system structures. LHX4 has gained much attention, as it has been found to be crucial for pituitary development [6]. In mice, *Lhx4* is expressed in the cerebral cortex, spinal cord, developing hindbrain [9] and pineal gland [10], and it plays an important role in the differentiation of ventral motor neurons [11]. In the developing pituitary gland, *Lhx4* is necessary for the regular differentiation of the pituitary cell types [7]. Eventually, as the AP develops, the expression of *Lhx4* in the gland declines and is completely absent in differentiated cells. However, its expression has also been documented in cells featuring stem/progenitor cell characteristics among the developed pituitary [12].

Human patients heterozygous for a mutation in LHX4 protein suffer from combined pituitary hormone deficiency (CPHD), short statures, reproductive and metabolic disorders and abnormalities of the sella turcica [6,8,9,13]. Unlike human patients, heterozygous *Lhx4*-mutant mice display no abnormalities [8]. However, in homozygous *Lhx4*-mutant mice, the pituitary begins to develop but later the AP cells undergo massive apoptosis, leading to AP hypocellularity. Homozygous *Lhx4*-mutant mice die shortly after birth due to severe lung defects [14], limiting research on the role of *Lhx4* in mice.

An alternative animal model to study pituitary development and AP cell determination is the zebrafish. This species is particularly attractive owing to the large number of accessible transparent progeny produced in each cross and their amenability to genetic manipulation and superb real-time imaging. Moreover, the rapid development of the zebrafish AP has been extensively studied. As in mammals, the zebrafish AP originates in the anterior neural ridge (ANR). At 18 h post-fertilization (hpf), the ANR thickens, and the developing AP begins invagination. Throughout the early stages of zebrafish development, the AP migrates posteriorly from the ANR, and it reaches its final position at 60 hpf [15]. As early as 24 hpf, *prl* and *gh* mRNAs can already be detected in the AP. The expression of glycoprotein hormone alpha-subunit (*αgsu*) starts at 32 hpf, and the expressions of *tshb* and *fshb* initiate at 42 hpf and 4 days post-fertilization (dpf), respectively [15]; *lhb* expression begins only later, at 25 dpf [16]. *Gh* is fully expressed by somatotrophs at 48 hpf [15]. The expression of *lhx4* in the adenohypophyseal placode, as well as in the pineal gland and trigeminal ganglion, is detected prior to those of most pituitary hormones [17,18]. Thus, utilization of the zebrafish model may further contribute to understanding the role of *LHX4* in AP development and the outcomes of LHX4 deficiency [19].

Here, we generated and characterized a zebrafish *lhx4*-knockout (KO) line to investigate the role of *lhx4* in pituitary gland development and functioning. Importantly, unlike mice, *lhx4*-KO fish survive and reach maturity. We discovered that at the embryonic and larval stages, *lhx4* mutants produce lower levels of *tshb* mRNA. At later stages, *lhx4* mutants also display decreased *gh*, *tshb*, *pomca* and *fshb* mRNA levels, undeveloped *lhb*-producing gonadotrophs and a reduced *pomca* promoter-driven expression in corticotrophs, along with smaller bodies and reproductive deficiencies, phenotypes that are reminiscent of human CPHD.

## 2. Results

### 2.1. Generation of Lhx4-Deficienct Zebrafish

**Figure 1:**
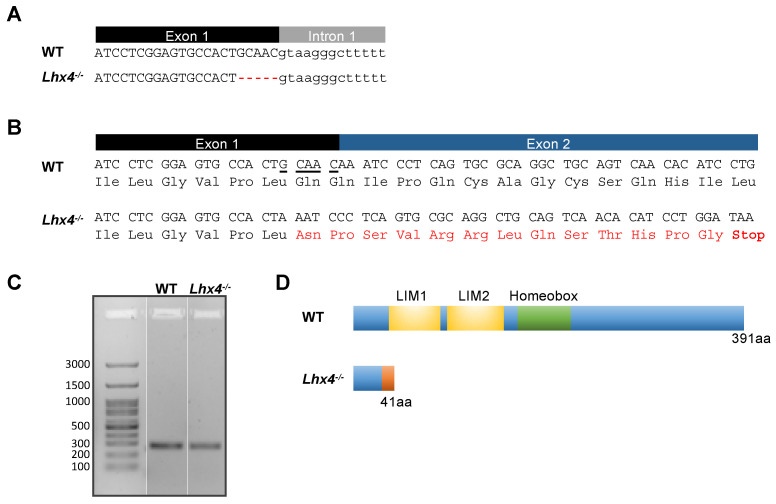
Characterization of the *lhx4* mutation. (**A**) A 5 bp deletion mutation (denoted by red dashes) at the end of exon 1 of the *lhx4* gene was generated by the CRISPR-Cas9 system. (**B**) The cDNA sequences derived from WT and *lhx4*-mutant brains indicate a frameshift caused by the deletion mutation (underlined in WT sequence), leading to 13 altered amino acids (aas) (red) and an early stop codon in exon 2. (**C**) Gel Electrophoresis of PCR products amplified from WT and *lhx4*-KO cDNAs using a primer set targeting exons 1 and 3 yielded a 265 bp WT and 260 bp mutant product. The similar product lengths confirm that the splicing of the *lhx4* mRNA was not altered by the mutation. (**D**) The *lhx4* mutation resulted in a predicted 41 aa truncated protein (bottom), including altered aas (orange), as compared with the 391 aa WT protein (top). The positions of the LIM1 and LIM2 domains (yellow) and homeobox domain (green) are designated in the WT protein.

Zebrafish *lhx4* is located in chromosome 8 and consists of six exons [20]. Using the CRISPR-Cas9 system, we generated an allele of *lhx4* with a five-base-pair (bp) deletion at the 3′ end of the first exon (Figure 1A). The deletion resulted in a frameshift, leading to the integration of an early stop codon in exon 2 (Figure 1B). A sequence analysis of mRNA extracted from the mutants’ brains reveals that even though the deletion is positioned at the end of exon 1, the splicing of exons 1 and 2 was not altered by the mutation (Figure 1C). Hence, the mutation results in a predicted truncated Lhx4 protein, lacking the LIM and homeobox domains (Figure 1D).

### 2.2. Pituitary Tshb Expression Is Affected by Lhx4 Deficiency at the Embryonic and Larval Stages

**Figure 2:**
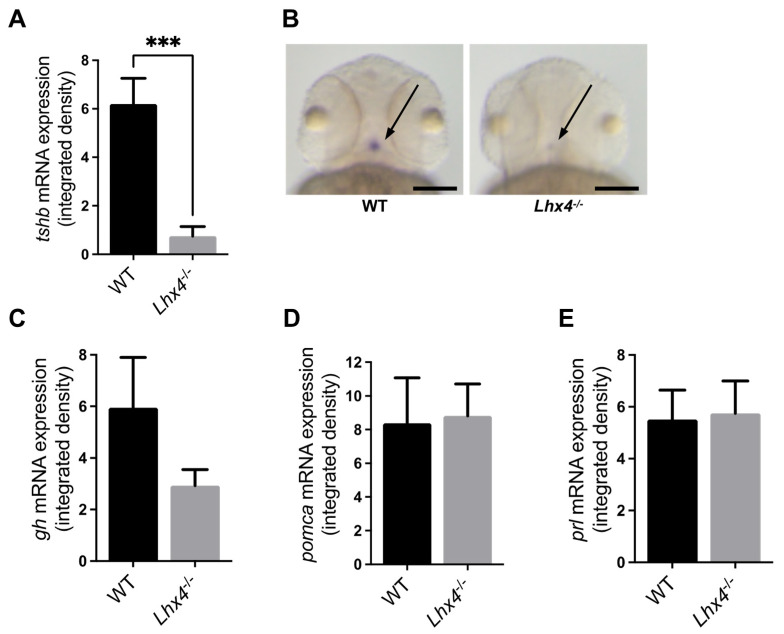
Whole-mount ISH analysis of pituitary hormone-encoding transcripts in 48 hpf embryos. (**A**,**C**–**E**) Transcript levels measured by the integrated densities of the whole-mount-ISH-staining signals of *tshb* ((**A**); N = 10 homozygotes and 13 WTs), *gh* ((**C**); N = 18 homozygotes and 10 WTs), *pomca* ((**D**); N = 13 homozygotes and 11 WTs) and *prl* ((**E**); N = 10 homozygotes and 6 WTs) in 48 hpf *lhx4* mutants and their WT siblings from heterozygous intercross. Homozygous *lhx4* mutants expressed lower amounts of *tshb* compared with their WT siblings ((**A**); *** *p* < 0.001, Mann–Whitney test), while the expressions of *gh*, *pomca* and *prl* remained unaltered (**C**–**E**). Error bars indicate s.e.m. (**B**) Representative samples (heads of 48 hpf embryos, dorsal view) of WT sibling (**left**) and homozygous *lhx4* mutant (**right**), analyzed by whole-mount ISH using the *tshb* probe. *Tshb* ISH signal (denoted by arrows) is substantially reduced in the pituitary of homozygous *lhx4* mutant at 48 hpf as compared with its WT sibling. Scale bar = 100 µm.

Since *lhx4* is expressed in the AP [17,18] (Figure S1), we sought to explore the effect of Lhx4 deficiency on the expressions of various hormones secreted by the AP cells. For this purpose, we examined the expression levels of *tshb*, *gh*, *pomca* (proopiomelanocortin, the ACTH precursor) and *prl* mRNAs in 48 hpf *lhx4* mutants and their wildtype (WT) siblings by whole-mount in situ hybridization (ISH) analysis. The expression of *tshb* was found to be considerably lower in the pituitary of the homozygous *lhx4* mutants in comparison with that of their WT siblings (Figure 2A,B). Nevertheless, no significant differences in the expressions of *gh*, *pomca* and *prl* were observed at this stage (Figure 2C–E).

**Figure 3:**
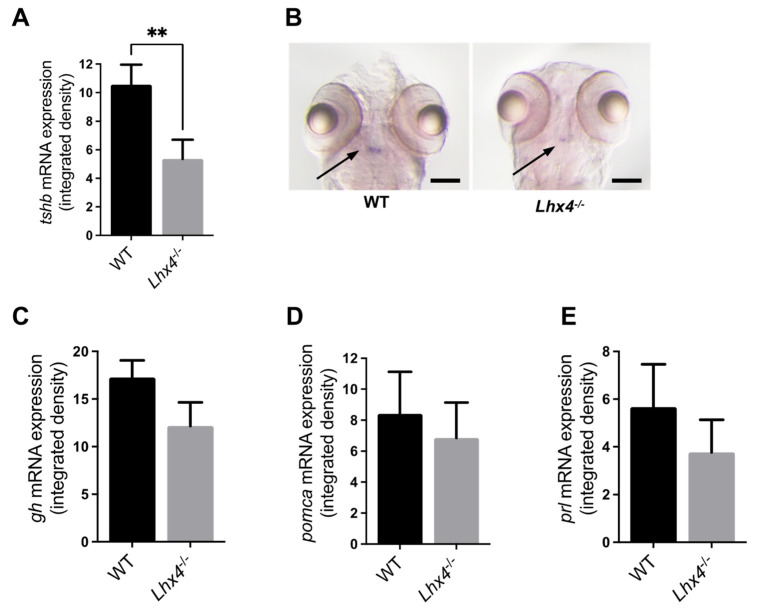
Whole-mount ISH analysis of pituitary hormone-encoding transcripts in 7 dpf larvae. (**A**,**C**–**E**) Transcript levels measured by the integrated densities of whole-mount-ISH-staining signals of *tshb* ((**A**); N = 10 homozygotes and 10 WTs), *gh* ((**C**); N = 11 homozygotes and 10 WTs), *pomca* ((**D**); N = 9 homozygotes and 4 WTs) and *prl* ((**E**); N = 9 homozygotes and 9 WTs) in 7 dpf *lhx4* mutants and their WT siblings from heterozygous intercross. Homozygous *lhx4* mutants expressed lower amounts of *tshb* compared with their WT siblings ((**A**); ** *p* < 0.01, Mann–Whitney test), while the expressions of *gh*, *pomca* and *prl* remained unaltered (**C**–**E**). Error bars indicate s.e.m. (**B**) Representative samples (heads of 7 dpf larvae, dorsal view) of WT sibling (**left**) and homozygous *lhx4* mutant (**right**), analyzed by whole-mount ISH using the *tshb* probe. *Tshb* ISH signal (denoted by arrows) is considerably reduced in the pituitary of homozygous *lhx4* mutant at 7 dpf as compared with its WT sibling. Scale bar = 100 µm.

*Tshb* expression in zebrafish can be detected starting from 42 hpf [19]. However, T3—the active molecule produced from T4 which is secreted by the thyroid gland as a result of *tshb* stimulation—is maternally transferred into the egg yolk [21]. This maternal T3 can exert a negative feedback on the hypothalamus and the AP, hence affecting the expression of *tshb* [22]. Therefore, we repeated the analysis in 7 dpf larvae, minimizing the potential effect of maternal T3 on the expressions of AP hormone-coding genes. Again, we found that of all the tested pituitary hormone-coding genes, only the *tshb* expression was altered by Lhx4 deficiency at the larval stage (Figure 3).

Since the hypothalamus–pituitary–thyroid (HPT) axis affects the metabolic rate, we examined the locomotor activity of the *lhx4* mutants, as a proximal index of metabolism [23]. For this purpose, *lhx4*-mutant larvae and their WT siblings were tested for their basal locomotor activity throughout 4 h of monitoring at 9 dpf. We found that the homozygous *lhx4* mutants were less active than their WT siblings (Figure S2A), and that their maximal speed was reduced (Figure S2B), supporting our assumption that the HPT axis, and, accordingly, metabolism, are interrupted by Lhx4 deficiency.

### 2.3. Thyroid Hormone Production at the Larval Stage Is Not Affected by Lhx4 Deficiency

The results presented above suggest a disruption of the HPT axis in the *lhx4* mutants. Since THs are directly regulated by TSHs, we aimed to examine the effect of *lhx4* KO on the TH production. To achieve this, we measured the T3 and T4 levels in 7 dpf *lhx4*-mutant larvae and their WT siblings by whole-mount immunohistochemistry. No significant differences in the TH production were detected between the homozygous *lhx4* mutants and their WT siblings at this age (Figure S3). This observation is consistent with an earlier study showing that an effect of *tshb* KO on the T4 and T3 levels in zebrafish becomes evident only after 20 and 25 dpf, respectively [22].

### 2.4. Impaired Growth of lhx4 Mutants

**Figure 4:**
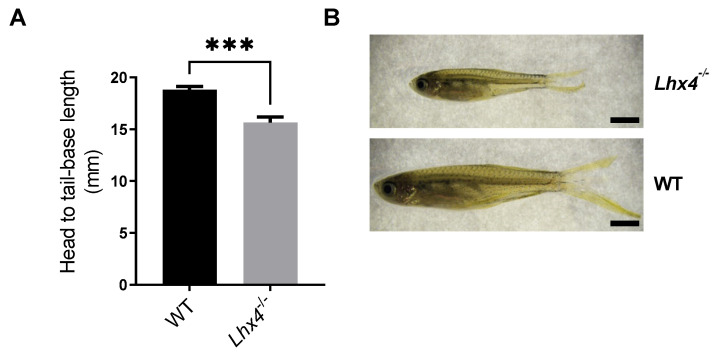
Reduced body size of adult *lhx4* mutants. (**A**) Bar chart representing head-to-tail-base length (mm) of 5-month-old *lhx4*-mutant fish and WT siblings that were mutually raised under controlled conditions. Homozygous *lhx4* mutants (N = 42) are significantly shorter than their WT siblings (N = 59; *** *p* < 0.001, Mann–Whitney test). Error bars indicate s.e.m. The presented results were pooled from four independent repeats. (**B**) Representative adult homozygous *lhx4* mutant (**top**) and WT sibling (**bottom**), lateral views. Bar scale = 2 mm.

As previously described, LHX4 deficiency affects the development and functioning of the mammalian pituitary, leading to a short stature in humans [6,8,9,13]. Based on our findings that *lhx4*-KO embryos and larvae express reduced *tshb* levels, and that their general locomotor activity is dampened, we set out to explore the effects of the *lhx4* mutation on their growth. To this end, *lhx4* mutants and their WT siblings were mutually raised under controlled conditions, and their growth was measured at 5 months of age. We found that the homozygous *lhx4* mutants were significantly smaller than their WT siblings (Figure 4), reproducing the mammalian phenotype.

### 2.5. Pituitary Hormone Deficiency in lhx4 Mutants

**Figure 5:**
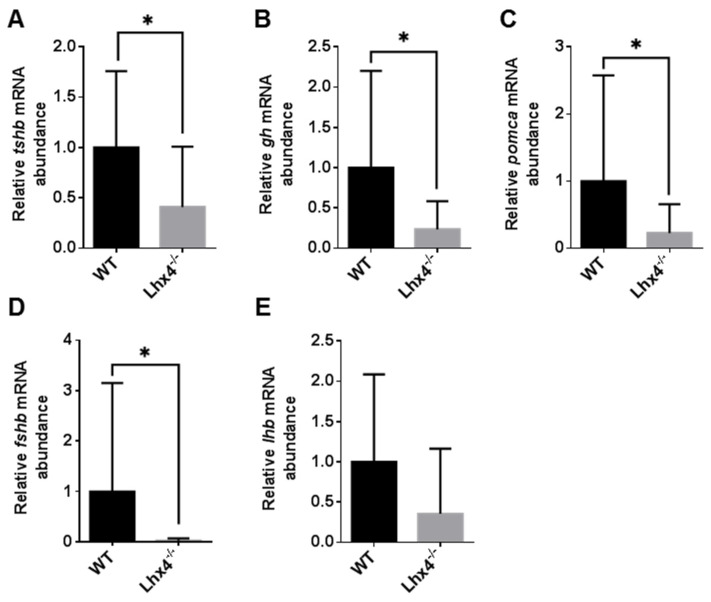
qRT-PCR analysis of pituitary hormone-encoding transcripts in adult fish. The relative expressions of pituitary hormone-coding mRNAs in pituitary glands dissected from 4-month-old *lhx4* mutants (N = 13) and their WT siblings (N = 10), as measured by qRT-PCR. Adult homozygous *lhx4* mutants express significantly lower mRNA levels of *tshb* (**A**), *gh* (**B**), *pomca* (**C**) and *fshb* (**D**) in the pituitary, compared with their WT siblings (* *p* < 0.05, Mann–Whitney test), while the *lhb* mRNA levels are insignificantly reduced (**E**). Error bars indicate s.e.m.

In view of the reduced growth of *lhx4*-mutant fish and the reduced expression of *tshb* during the embryonic and larval stages, we aimed to measure the expression levels of genes encoding AP hormones also at the adult stage. We utilized the Tg(*pomca*:GFP) reporter line [24] to facilitate the isolation of the small pituitary gland without the contamination of the surrounding tissue. Tg(*pomca*:GFP);*lhx4*-KO fish and control Tg(*pomca*:GFP) siblings were obtained by crossings and raised to adulthood. At 4 months of age, their pituitaries were dissected, and the transcript levels were evaluated by quantitative real-time PCR (qRT-PCR) analysis. Consistent with our observations at the embryonic and larval stages, the adult *lhx4* mutants expressed significantly lower levels of *tshb* mRNA compared with their WT siblings (Figure 5A). Furthermore, the *gh* and *pomca* mRNA levels were also reduced in the adult *lhx4* mutants (Figure 5B,C), in contrast to our observations at the younger stages. As for gonadotropins, the *fshb* expression was reduced in the pituitaries of the adult *lhx4* mutants (Figure 5D), while the *lhb* mRNA levels were considerably, yet insignificantly, lowered (Figure 5E). The reduced expressions of most of the pituitary gland hormones in the *lhx4*-KO fish recapitulate the phenotype of human CPHD.

### 2.6. Corticotroph Abnormality in Lhx4-Deficient Fish

**Figure 6:**
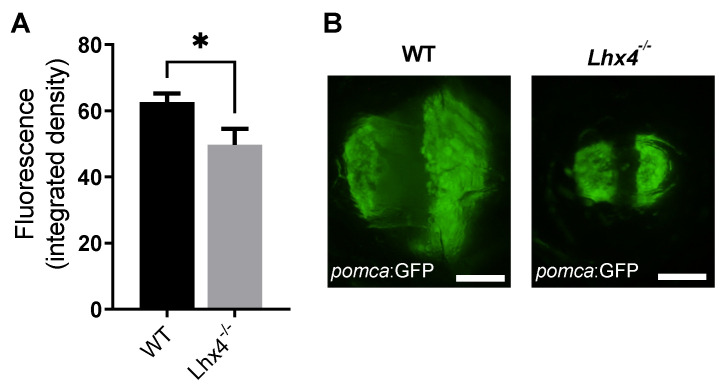
Reduced pomca-driven expression in corticotrophs of adult *lhx4* mutants. (**A**) Tg(*pomca*:GFP) reporter line was utilized for evaluating corticotroph development in 4-month-old *lhx4* mutants (N = 21) and their WT siblings (N = 18). The integrated fluorescence in the pituitaries of homozygous *lhx4* mutants was reduced (* *p* < 0.05, *t*-test) as compared with that of their WT siblings. (**B**) Representative *pomca* promoter-driven fluorescence in the pituitaries of adult WT (**left**) and homozygous *lhx4* mutant (**right**). Scale bar = 100 µm.

To assess the effect of Lhx4 deficiency on the corticotroph development, we monitored the GFP expression under the pomca promotor in the pituitaries of mutually raised Tg(*pomca*:GFP);*lhx4*-KO fish and their control Tg(*pomca*:GFP) siblings at the age of 4 months. The extent of the GFP fluorescence in the pituitaries of the *lhx4* mutants was decreased compared with that of their control siblings (Figure 6), corresponding to our finding of reduced *pomca* mRNA levels in adult *lhx4* mutants, and implying that corticotroph development is impaired by the *lhx4* mutation.

### 2.7. Reproductive Failure and Impaired Gonadotroph Development in Lhx4-Deficient Females 

*Tshb* mutant zebrafish have been shown to be infertile [22]. Infertility has also been reported in *lhb*-mutant zebrafish females [25]. Since *lhx4* mutants express lower *tshb* mRNA levels at both the early and adult stages, and reduced levels, albeit insignificant, of *lhb* mRNA at the adult stage, we set out to examine the reproductive success of *lhx4* mutants. Throughout the study, we observed that while the homozygous *lhx4*-mutant males were able to reproduce when crossed with WT females, the homozygous *lhx4*-mutant females did not produce eggs in any type of cross.

**Figure 7:**
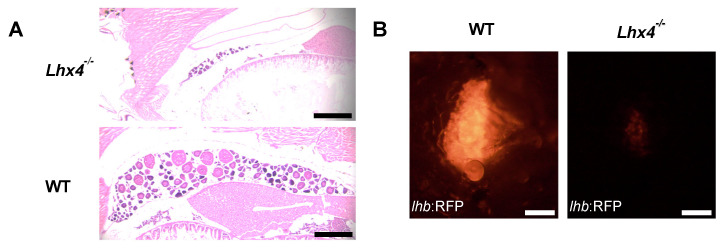
Immature ovaries and depleted gonadotrophs in *lhx4* KO females. (**A**) Hematoxylin and eosin histology performed on adult fish ovary sections, demonstrating an undeveloped ovary of homozygous *lhx4*-mutant female (**top**), compared with a properly developed WT sibling ovary (**bottom**). Scale bar = 200 µm. (**B**) Expression of RFP under the tilapia *lhb* promoter in the pituitary of adult WT sibling female (**left**) and homozygous *lhx4*-mutant female (**right**), indicating severely impaired gonadotroph development in the mutant. Scale bar = 100 µm.

To evaluate the oocyte development, we examined the ovaries of adult WT and homozygous *lhx4*-mutant females by hematoxylin and eosin (H&E) histology. As expected, the ovary and follicle sizes of the *lhx4*-mutant females were reduced compared with those of their WT siblings (Figure 7A).

Accordingly, we further evaluated the effects of Lhx4 deficiency on *lhb*-expressing gonadotrophs. For this purpose, we utilized the Tg(*lhb*:RFP) reporter line [5], which expresses RFP under the tilapia (Oreochromis niloticus) *lhb* promoter, and which generated Tg(*lhb*:RFP);*lhx4*-KO fish and control WT Tg(*lhb*:RFP) siblings, which were mutually raised under controlled conditions. In accordance with the observed female infertility, the RFP intensity in the *lhb*-expressing gonadotrophs of the homozygous *lhx4*-mutant females was nearly undetectable (Figure 7B).

## 3. Discussion

Various dominant LHX4 mutations have been discovered in humans, which has led to a variety of phenotypes in heterozygous carriers, such as a short stature due to GH deficiency, CPHD, abnormalities of the central skull base and cerebellar defects [8,26]. A recessive mutation in LHX4 has also been reported. The heterozygous parents were unaffected by the mutation; however, their three homozygous children were born underweight, suffered from poor muscle tone, had severe lung abnormalities, and died within the first week after birth. ACTH, TSH and GH deficiencies were diagnosed in these infants [26]. Similar to heterozygous humans carrying a recessive LHX4 mutation, heterozygous *Lhx4*-mutant mice exhibit no apparent phenotype [9,26]. Homozygous *Lhx4*-mutant mice successfully develop the Rathke-pouch ectoderm structure but have a severely hypoplastic AP [27]. They die shortly after birth due to lung defects, hindering studies on the role of *Lhx4* in the pituitary function at later stages [14].

The *lhx4*-KO zebrafish generated in this study (Figure 1) expressed lower levels of *tshb* mRNA at 48 hpf, 7 dpf and 4 months of age (Figure 2A,B, Figure 3A,B and Figure 5A, respectively). Although no differences were found in the expressions of *gh*, *pomca* and *prl* at the early stages (Figure 2C–E and Figure 3C–E), the expressions of *gh* and *pomca* were significantly reduced in the adult *lhx4* mutants (Figure 5B,C). The development of corticotrophs was also affected by Lhx4 deficiency, as indicated by the reduced *pomca* promoter-driven GFP expression in the pituitaries of adult mutants (Figure 6). Decreased *fshb* expression was observed in the adult homozygous *lhx4* mutants (Figure 5D). Moreover, utilizing a transgenic reporter line in which the expression of RFP is driven by the tilapia *lhb* promoter, we have shown that *lhb*-expressing gonadotrophs are severely depleted in the pituitaries of adult *lhx4* mutants (Figure 7B).

Owing to the survival of *lhx4*-KO zebrafish and the fact that they reach the adult stage, the phenomenon of the sequential loss of the pituitary hormone-producing cells could be observed. This phenomenon could be explained by the effect of LHX4 deficiency on pituitary precursor cells: In *lhx4*-mutant mouse embryos, increased levels of apoptotic pituitary precursor cells have been documented, indicating that *Lhx4* is necessary for the survival of precursor cells and thereby controls the number of differentiated pituitary hormone-secreting cells [28]. Likewise, it is possible that a population of stem cells within the adult zebrafish AP, as is the case with mouse embryos [12], is depleted in Lhx4-deffienct fish, resulting in the reduced proliferation of hormone-secreting cells in the AP. Alternatively, the sequential decreased expressions of pituitary hormone-encoding genes may be an indirect outcome of Lhx4 deficiency. The finding that the *tshb* expression is reduced in *lhx4* mutants at the embryo and larval stages (Figure 2A,B and Figure 3A,B), prior to the observed decrease in other pituitary hormone-encoding genes, implies that at least part of the adult phenotype may be caused by TH deficiency, which is expected to occur only at later life stages, 20 dpf [22]. Since THs are known to regulate growth, in addition to metabolism and reproduction [2,22,23], the substantially lower expression of *tshb* in the homozygous *lhx4* mutants may account for their small size (Figure 4) and immature gonads (Figure 7A). Notably, the levels of the mediators of these effects, THs, were not altered by the mutation at 7 dpf (Figure S3). However, as indicated above, the effect of TSH deficiency on TH signaling is not expected at this stage [22], and the maternal TH deposited in the yolk, in both WT and *lhx4* mutants, is sufficient for the initiation of the normal development of the nervous system [21]. 

An additional explanation for the reduction in the pituitary cell types in adult Lhx4-deficient zebrafish could be reduced innervation or decreased blood flow to the gland. This assumption is based on the fact that *LHX4* mutations in humans lead to a reduction in the size of the pituitary stalk [29]. Furthermore, *lhx4* is also expressed outside the AP [8,11,17,30] and (Figure S1), the phenotypes described here may be induced by pathways that are unrelated to the pituitary function. For example, the reduced locomotor activity of the *lhx4*-mutant larvae (Figure S2) could have stemmed from poor muscle tone, as is the case in human patients [26]. Thus, the poor mobilization of the *lhx4* mutants may have led to a failure in the competition over food when raised with their WT siblings, which may account for their malnutrition and reduced body size [31], a possibility that warrants further inquiry.

The reproductive impairments of the homozygous *lhx4*-mutant females could also be explained by malnutrition and a reduced body size, although our findings of lower *fshb* mRNA levels and depleted *lhb*-expressing gonadotrophs in adult homozygous *lhx4* mutants would be a more reasonable explanation [25,32]. Thus, we conclude that gonadotropin deficiency, or the combination of gonadotropin and Tshb deficiencies, is the source for the female infertility of homozygous *lhx4*-mutant females. As opposed to the females, the *lhx4*-mutant males were fertile, indicating that the reduced pituitary hormone levels have a stronger effect on ovarian development in comparison with testicular development. This implies that the combined hormonal profile required for ovarian development and vitellogenesis, absent in the *lhx4* mutant, is different from that required for testicular development. An alternative explanation could be a direct effect of Lhx4 on gonadal development: In mice, an RNAseq analysis of developing gonads revealed a significantly higher expression of *Lhx4* mRNA in the developing ovary compared to that in the developing testes [33], possibly explaining the sex-specific effect of LHX4 deficiency in zebrafish. This could be an interesting avenue to investigate despite the apparent differences between the development of mammalian and fish reproductive systems.

In summary, our *lhx4*-KO model exhibits phenotypes that resemble those observed in human patients carrying a LHX4 mutation, such as CPHD, impaired growth and fertility abnormalities. Unlike other models, *lhx4*-mutant zebrafish survive the larval stage and reach adulthood. We found that although *lhx4* is mainly expressed in the developing AP, it exerts its function throughout all the zebrafish life stages. Hence, the characterized *lhx4*-mutant line constitutes a valuable model to further investigate the consequence of *lhx4* mutation on pituitary development, pituitary functioning and beyond. Further research is required to define the cellular, temporal and spatial expression of *lhx4* in the zebrafish AP, and to understand how this expression pattern affects the various AP cell types and the physiological processes they regulate.

## 4. Materials and Methods

### 4.1. Fish and Embryos

Zebrafish (Danio rerio) were grown and maintained in a recirculating-water system at 28 °C under 12:12 h LD cycles and fed twice a day. The fish were naturally mated in an appropriate tank, and the embryos were collected and kept in a Petri dish with embryo water containing methylene blue (0.3 ppm) in an incubator at 28 °C under 12:12 h LD cycles. On the 7th day, larvae were transferred to 10 L tanks in the recirculating-water system. Once the fish reached adulthood, they were genotyped and transferred accordingly into 3 L tanks.

### 4.2. Generation of lhx4-Mutant Zebrafish and Genotyping

The CRISPR-Cas9 system was used to establish the *lhx4*-KO zebrafish line, registered in the Zebrafish Information Network (ZFIN) database as *lhx4^tlv12^*. Oligos 5′-taggagtgccactgcaacgtaa-3′ and 5′-aaacttacgttgcagtggcact-3′ were designed to target a sequence at the end of exon 1 (5′-GGAGTGCCACTGCAACgtaa-3′), which contains the BtsI-v2 restriction site (underlined). The oligos were ligated into a pT7-gRNA zebrafish-optimized vector (plasmid #46759, Addgene, Watertown, MA, USA), followed by linearization with BamHI (R3136, New England Biolabs, Ipswich, MA, USA), and the synthesis of gRNA was performed with a MAXIscript T7 Transcription Kit (AM1312, Invitrogen, Waltham, MA, USA). An injection mix was prepared by mixing gRNA (60 ng μL^−1^) and TrueCut Cas9 Protein V2 (1 μg μL^−1^; A36498, Invitrogen), followed by 5 min incubation in 37 °C prior to co-injection into one-cell-stage WT zebrafish embryos. Injected embryos (F0 generation) were raised to maturity and crossed with WT fish to identify carriers of an indel within the *lhx4* gene at the F1 generation.

For genotyping, whole larvae or fin samples from mature fish were lysed in lysis buffer [10 mM Tris (pH 8), 2 mM EDTA (pH 8), 0.2% Triton X-100 and 0.1 mg mL^−1^ protein kinase]. Fixated post-whole-mount ISH and immunostained samples were lysed using the Extract-N-Amp™ FFPE Tissue PCR kit (XNAT2-1KT, Sigma, St. Louis, MO, USA), according to the manufacturer’s protocol. Lysis was performed overnight at 52 °C, followed by 10 min inactivation at 95 °C. The isolated genomic DNA served as the template to amplify a 490 bp fragment of the *lhx4* gene using forward 5′-atgaaaatgatgcaaagtgcg-3′ and reverse 5′-tgcccagctatgcgatctaac-3′ primers. Identification of the *lhx4*-mutant allele was based on the incomplete digestion of the PCR product by BtsI-v2 (R0667, New England Biolabs, Ipswich, MA, USA), in contrast to the full digestion of the WT allele into two fragments (of 76 bp and 414 bp). Sequence analysis of the selected F1 founder genomic DNA indicated a 5 bp deletion at the end of *lhx4* exon 1, and the *lhx4^tlv12^* line was propagated by further crossings to produce homozygous mutants and WT siblings at future generations. 

### 4.3. Reverse Transcription PCR

RNA was purified from brain samples dissected from adult homozygous *lhx4* mutants and WT siblings using the RNeasy Lipid Tissue Mini kit (74804, Qiagen, Hilden, Germany). An amount of 1 µg of the purified RNA served as the template for the cDNA synthesis using the qScript cDNA Synthesis Kit (95047, Quantabio, Beverly, MA, USA). PCR was performed on cDNA templates using forward primer 5′-atgaaaatgatgcaaagtgcg-3′ targeting the beginning of exon 1, and reverse primer 5′-cgaaacgcttgaagaagtcc-3′ spanning the exon 2–3 junction, yielding a 265 bp product.

### 4.4. Whole-Mount In Situ Hybridization

An 815 bp fragment of the zebrafish *lhx4* (RefSeq NM_001122973.1) coding sequence (CDS) was amplified using forward 5′-ggacttcttcaagcgtttcg-3′ and reverse 5′-tcagagcttgacccacactg-3′ primers and cloned into pGEM-T Easy (A1360, Promega, Madison, WI, USA). In addition, plasmids containing CDS fragments of zebrafish *gh1* (RefSeq NM_001020492.2), *tshba* (RefSeq NM_181494.2), *prl* (RefSeq NM_181437.3) and *pomca* (RefSeq NM_181438.3) were kindly provided by The Hammerschmidt Plasmid Stocks (Spemann Labs, Freiburg, Germany). Plasmids were linearized, and digoxigenin (DIG)-labeled anti-sense riboprobes were synthesized using the Dig RNA Labeling Kit (SP6/T7; 11175025910, Roche, Basel, Switzerland), according to the manufacturer’s instructions.

The embryos/larvae were fixed at 24 and 48 hpf and 7 dpf, and whole-mount ISH was performed as previously described [34], with the following modification: the 24 and 27 hpf sampled embryos were not treated with proteinase K. Images were acquired (see Section 4.11), and the staining signal was quantified using ImageJ software 2.1.0. (National Institute of Health, Bethesda, MD, USA). The staining signal, presented as the integrated (optical) density, was computed by multiplying the area (pixels) by the mean intensity value. After image quantification, each embryo/larva was genotyped (see ‘Generation of *lhx4*-Mutant Zebrafish and Genotyping’). Statistical differences between genotypes were determined by Mann–Whitney test.

### 4.5. Whole-Mount Immunostaining 

The immunostaining protocol was carried out as published in [35], with minor adjustments. In short, 7 dpf fixed larvae were incubated in 10% H_2_O_2_ at room temperature for 4 h and then washed 3 times with PBT solution (0.25% Triton X-100 in PBS). The samples were blocked for 2 h at room temperature with 4% blocking solution (containing donkey serum), followed by overnight incubation at 4 °C with primary antibodies: anti-mouse triiodothyronine (T3) (1:100; ME-124, sc-57481, Santa Cruz Biotechnology, Dallas, TX, USA) or anti-rabbit thyroxine (T4) (1:100; 8658501, MP bio, Irvine, CA, USA).

After 6 washes with PBT, larvae were incubated for 4 h with secondary antibodies: donkey anti-mouse Alexa Fluor 488 (1:500; 715-545-150, Jackson ImmunoResearch, West Grove, PA, USA) or donkey anti-rabbit Cy3 (1:500; 711-165-152, Jackson ImmunoResearch, West Grove, PA, USA) for T3 and T4, respectively. Afterwards, the fluorescent signal was captured (see Section 4.11) and quantified as described for the whole-mount ISH signal (see Section 4.4). Subsequently, larvae were genotyped as described in ‘Generation of *lhx4* mutant zebrafish and genotyping’. Statistical differences between groups were evaluated by Mann–Whitney test.

### 4.6. Histology

Homozygous *lhx4*-mutant females (N = 2) and their WT siblings (N = 6) at the age of 4 months were fixed in 4% PFA. After decalcification and paraffin embedding, longitude sections (4 µm) were prepared and stained with H&E by Gavish Research Services. Ovary slide images were acquired (see Section 4.11).

### 4.7. Larval Locomotor Activity Assay

Progeny of heterozygous *lhx4*-mutant intercross were raised in an incubator under 12:12 h LD cycles. At 9 dpf, the larvae were individually placed in wells of a 24-well plate in the observation chamber of the DanioVision tracking system (Noldus Information Technology, Wageningen, The Netherlands). The activity of each larva was tracked for 4 h under constant light and analyzed by Ethovision 15.0 software (Noldus Information Technology, Wageningen, The Netherlands) for the total activity (log_cm_) and top speed (cm s^−1^). Following activity monitoring, larvae were lysed and genotyped as described (see ‘Generation of *lhx4*-Mutant Zebrafish and Genotyping’). Statistical differences between genotypes were determined by *t*-test with Benjamini–Hochberg correction for multiple comparisons to maintain a false discovery rate of 0.05.

### 4.8. Body Size Measurement

To quantify the body size, adult homozygous *lhx4* mutants and their siblings at the age of 5 months were anesthetized with 0.16 mg mL^−1^ tricaine (A-5040, Sigma), laterally placed on a Petri dish plate and photographed (see Section 4.11). The body size was evaluated as the distance from the head to the tail-base, using an in-house custom RStudio version 2023.09.1+494 script. Statistical differences between genotypes were determined by Mann–Whitney test.

### 4.9. Quantitative Real-Time RT-PCR Analysis

Pituitary glands were dissected from homozygous *lhx4* mutants (N = 10) and their WT siblings (N = 13), and RNA was extracted using the RNeasy Micro Kit (74004, Qiagen, Hilden, Germany), according to the manufacturer’s instructions. cDNA was synthesized with the qScript cDNA Synthesis Kit (95047, QuantaBio, Beverly, MA, USA). qRT-PCR was carried out using the following primer sets: *tshba* (RefSeq NM_181494.2): forward 5′-cccccactgactacaccatctac-3′ and reverse 5′-gcatcccctctgaacaataaaacgag-3′ primers yielding a 149 bp product; *gh1* (RefSeq NM_001020492.2): forward 5′-gctgcttcgtatctctttccgcc-3′ and reverse 5′-ggctgtccatcgagacatccc-3′ primers yielding a 174 bp product; *pomca* (RefSeq NM_181438.3): forward 5′-cgagcaaacgcaaagacaac-3′ and reverse 5′-gccaagcaggacacaacatc-3′ primers yielding a 121 bp product; *fshb* (RefSeq NM_205624.1): forward 5′-ggactatgctggacaatggatcg-3′ and reverse 5′-tcagagccacggggtac-3′ primers yielding a 154 bp product; *lhb* (RefSeq NM_205622.2): forward 5′-acggtatcggtggaaaaagagg-3′ and reverse 5′-tacgtgcacactgtctggtg-3′ primers yielding a 134 bp product. The reference gene used for calculating the relative expression was *actb2* (RefSeq NM_181601.5), using forward 5′-ccccaaacccaagttcagcc-3′ and reverse 5′-acccacgatggatgggaaga-3′ primers that yielded a 128 bp product.

The qRT-PCR was performed using PerfeCTa SYBR green FastMix (95074-250-2, QuantaBio, Beverly, MA, USA) in a QuantStudio 1 instrument (Thermo Fisher Scientific, Waltham, MA, USA) and analyzed by QuantStudio^TM^ Design & Analysis Software v1.5.1. The qRT-PCR amplification protocol consisted of 20 s of initial denaturation at 95 °C, followed by 40 cycles of 1 s denaturation at 95 °C, annealing and extension at 60 °C for 20 s and a final melting-curve stage. The reactions were performed in triplicates and the relative gene expression was calculated by the comparative-threshold-cycle method (2^−∆∆Ct^). The WT expression was set to 1, and the gene expression of the *lhx4* mutant compared to that of the WT was calculated. Statistical differences in gene expression between genotypes were determined by Mann–Whitney test.

### 4.10. Transgenic Reporter Lines

Tg(−1.*0pomca*:GFP)^zf44^ [24] and Tg(Oni.*lhb*:TagRFP,myl7:TagRFP) [5] reporter lines were utilized; the latter also expresses RFP in heart cells for the detection of positive transgenic larvae, as *lhb* expression initiates only at a later stage. For accurate fluorescence-level comparisons, only reporter fish harboring a single transgenic insertion of GFP/RFP were used. The transgenic reporter lines and homozygous *lhx4* mutants were crossed, yielding heterozygous *lhx4* mutants. GFP/RFP-positive progeny were raised to adulthood and crossed with heterozygous *lhx4* mutants to produce homozygous *lhx4* mutants and WT siblings with a single transgenic allele.

When reaching maturity, fish were sacrificed, the pituitary was exposed by removing the jaws and the fluorescence was documented (see Section 4.11). Subsequently, fish were genotyped as previously described (see ‘Generation of *lhx4*-Mutant Zebrafish and Genotyping’). The mean intensity and area (pixels) of the GFP fluorescence were computed using ImageJ software 2.1.0. (National Institute of Health, Bethesda, MD, USA) and multiplied to produce the integrated density, and differences between genotypes were analyzed by *t*-test.

### 4.11. Imaging

Images were taken with an SZX16 Research Stereo Microscope (Olympus, Waltham, MA, USA) equipped with a camera (DP74) and cellSens Entry 2.1 software, using an Oblique high-contrast cartridge (SZX2-COBH). The X-Cite Xylis Broad Spectrum LED Illumination System (Excelitas technologies, Waltham, MA, USA) was used for fluorescence excitation.

The immunostaining signal was captured with an AX10 fluorescence microscope (Zeiss, Oberkochen, Germany) equipped with a camera and Zen 2.3 lite software.

## Data Availability

The original contributions presented in this study are included in the article/Supplementary Materials, and further inquiries can be directed to the corresponding author.

## Supplementary Materials

The following supporting information can be downloaded at: https://www.mdpi.com/article/10.3390/ijms25137332/s1.
