# Supplementary material for: Combined Pituitary Hormone Deficiency in lhx4-Knockout Zebrafish
Source: Int J Mol Sci. 2024 Jul 4;25(13):7332. doi: 10.3390/ijms25137332 (PMC11242037; doi:10.3390/ijms25137332)
Supplement: Supplementary file 1 [file ijms-25-07332-s001.zip › ijms-3064002-supplementary.pdf]

International Journal of
Molecular Sciences

MDPI

Supplementary Materials:

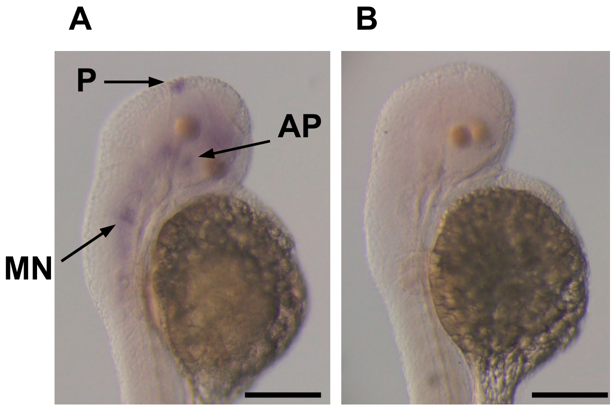

Figure S1. Spatiotemporal expression of *lhx4* in 48 hpf WT zebrafish. Whole-mount ISH staining of *lhx4* in 48 hpf WT zebrafish. **A)** *Lhx4* is expressed in motor-neurons (MN) in the spinal cord, pineal gland (P) and anterior pituitary (AP) (denoted by arrows). **B)** Negative control of whole-mount ISH staining with the sense probe of *lhx4*. Scale bar = 200 μm.

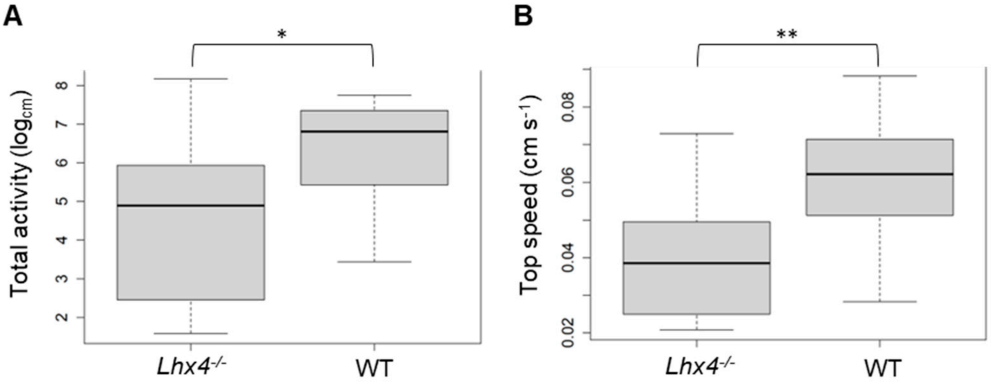

Figure S2. Reduced locomotor activity of *lhx4* mutant larvae. Locomotor activity of homozygous *lhx4* mutant larvae (N = 9) and their WT siblings (N = 13) was continuously monitored for 4 hours at 9 dpf. Boxplots present medians (solid lines) and interquartile ranges (gray boxes) within each group. **A**) Total activity (log_{cm}) of homozygous *lhx4* mutant larvae is significantly reduced in comparison with their WT siblings, *p< 0.05, t-test with Benjamini-Hochberg correction (false discovery rate = 0.05). **B**) The maximal speed (cm sec^{-1}) of homozygous *lhx4* mutant larvae is significantly reduced compared with their WT siblings, **p< 0.01, t-test with Benjamini-Hochberg correction (false discovery rate = 0.05).

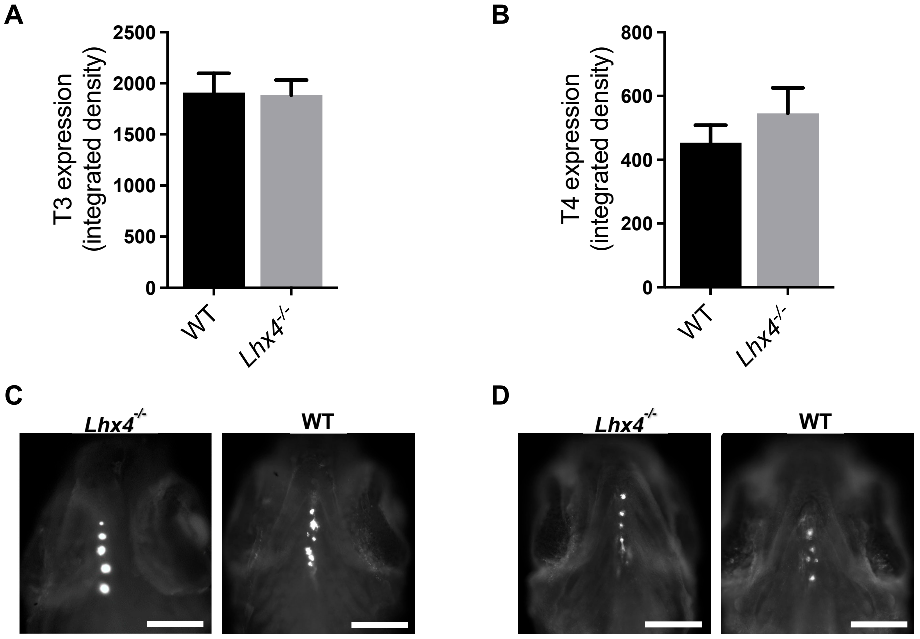

Figure S3. Unaltered levels of thyroid hormones in the thyroid of 7 dpf *lhx4* KO larvae. Levels of T3 (A) and T4 (B) in the thyroid of 7 dpf larvae, analyzed by whole-mount immunohistochemistry (integrated density of the fluorescent signal). **A**) No differences were found in T3 levels between homozygous *lhx4* mutants (N = 13) and their WT siblings (N = 12). **B**) No differences were detected in T4 levels between homozygous *lhx4* mutants (N = 13) and their WT siblings (N = 12). Error bars indicate s.e.m. **C**) Representative samples (heads of 7 dpf larvae, ventral view) of homozygous *lhx4* mutant and WT sibling immunostained with an antibody against T3. **D**) Representative samples (heads of 7 dpf larvae, ventral view) of homozygous *lhx4* mutant and WT sibling immunostained with an antibody against T4. Scale bar = 200 μm.
